# Tetra­ethyl­ammonium l-malate 1.36-hydrate

**DOI:** 10.1107/S1600536808040348

**Published:** 2008-12-10

**Authors:** Mohd Basyaruddin Abdul Rahman, Khairulazhar Jumbri, Kamaliah Sirat, Reza Kia, Hoong-Kun Fun

**Affiliations:** a Department of Chemistry, Faculty of Science, Universiti Putra Malaysia, 43400 UPM Serdang, Selangor, Malaysia; b X-ray Crystallography Unit, School of Physics, Universiti Sains Malaysia, 11800 USM, Penang, Malaysia

## Abstract

The asymmetric unit of the title compound, C_8_H_20_N^+^·C_4_H_5_O_5_^−^·1.36H_2_O, contains two independent ion pairs, with similar conformations, and three water mol­ecules of crystallization, one water mol­ecule haing a site-occupancy factor of 0.721 (5). Intra­molecular O—H⋯O hydrogen bonds, involving the hydr­oxy groups and an O atom of each carboxyl­ate anion, generate five-membered rings involving *S*(5) ring motifs. In the crystal structure, mol­ecules are linked together by water mol­ecules through four-membered O—H⋯O—H⋯O—H inter­actions to form one-dimensional infinite chains along the *a* axis. Since the mol­ecules are also linked into one-dimensional infinite chains along the *b* axis, mol­ecular sheets parallel to the (001) plane are created. Overall, the crystal structure is stabilized by two intra­molecular O—H⋯O hydrogen bonds, nine inter­molecular O—H⋯O and ten C—H⋯O hydrogen bonds.

## Related literature

For hydrogen-bond motifs, see: Bernstein *et al.* (1995 ▶). For bond-length data, see: Allen *et al.* (1987 ▶). For related compounds, see, for example: Rahman *et al.* (2008 ▶); Allen *et al.* (2006 ▶); Jiang *et al.* (2008 ▶). For related literature, see: Anandha *et al.* (2008 ▶).
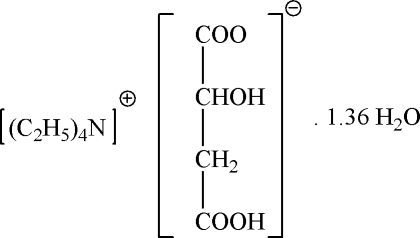


## Experimental

### 

#### Crystal data

C_8_H_20_N^+^·C_4_H_5_O_5_^−^·1.36H_2_O
*M* *_r_* = 287.83
Monoclinic, 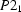

*a* = 7.4724 (2) Å
*b* = 19.9721 (5) Å
*c* = 10.2726 (3) Å
β = 92.481 (1)°
*V* = 1531.64 (7) Å^3^
*Z* = 4
Mo *K*α radiation
μ = 0.10 mm^−1^
*T* = 100.0 (1) K
0.45 × 0.35 × 0.32 mm

#### Data collection

Bruker SMART APEXII CCD area-detector diffractometer
Absorption correction: multi-scan (**SADABS**; Bruker, 2005 ▶) *T*_min_ = 0.950, *T*_max_ = 0.969
36497 measured reflections
8479 independent reflections
7551 reflections with *I* > 2σ(*I*)
*R*_int_ = 0.029

#### Refinement

*R*[*F* ^2^ > 2σ(*F* ^2^)] = 0.041
*wR*(*F* ^2^) = 0.103
*S* = 1.03
8479 reflections
373 parameters
1 restraint
H atoms treated by a mixture of independent and constrained refinement
Δρ_max_ = 0.51 e Å^−3^
Δρ_min_ = −0.47 e Å^−3^

### 

Data collection: *APEX2* (Bruker, 2005 ▶); cell refinement: *APEX2*; data reduction: *SAINT* (Bruker, 2005 ▶); program(s) used to solve structure: *SHELXTL* (Sheldrick, 2008 ▶); program(s) used to refine structure: *SHELXTL*; molecular graphics: *SHELXTL*; software used to prepare material for publication: *SHELXTL* and *PLATON* (Spek, 2003 ▶).

## Supplementary Material

Crystal structure: contains datablocks global, I. DOI: 10.1107/S1600536808040348/fj2170sup1.cif

Structure factors: contains datablocks I. DOI: 10.1107/S1600536808040348/fj2170Isup2.hkl

Additional supplementary materials:  crystallographic information; 3D view; checkCIF report

## Figures and Tables

**Table 1:** Hydrogen-bond geometry (Å, °)

*D*—H⋯*A*	*D*—H	H⋯*A*	*D*⋯*A*	*D*—H⋯*A*
O1*A*—H1O*A*⋯O4*A*^i^	0.82	1.68	2.4977 (11)	171
O3*A*—H3O*A*⋯O2*W*	0.82	1.98	2.7296 (14)	151
O3*A*—H3O*A*⋯O5*A*	0.82	2.27	2.6853 (11)	112
O3*B*—H3O*B*⋯O3*W*	0.82	2.00	2.7435 (13)	151
O3*B*—H3O*B*⋯O5*B*	0.82	2.26	2.6837 (12)	112
O1*W*—H1*W*1⋯O4*A*^ii^	0.92	2.03	2.9354 (17)	166
O1*W*—H2*W*1⋯O1*B*^iii^	0.92	1.90	2.8018 (18)	165
O2*W*—H1*W*2⋯O5*B*	0.84	1.99	2.7969 (13)	162
O2*W*—H2*W*2⋯O3*B*^iv^	0.72	2.18	2.8961 (13)	176
O3*W*—H2*W*3⋯O3*A*	0.80 (2)	2.13 (2)	2.9169 (13)	173 (2)
O3*W*—H1*W*3⋯O5*A*^i^	0.89 (2)	1.94 (2)	2.7894 (12)	160 (2)
C2*A*—H2*AB*⋯O1*W*^v^	0.97	2.44	3.3852 (18)	165
C5*A*—H5*AA*⋯O1*A*^ii^	0.97	2.41	3.2814 (15)	149
C6*A*—H6*AA*⋯O1*W*^i^	0.96	2.59	3.296 (2)	131
C6*A*—H6*AB*⋯O2*W*^i^	0.96	2.60	3.434 (2)	146
C7*A*—H7*AA*⋯O1*W*	0.97	2.42	3.2511 (18)	144
C11*A*—H11*B*⋯O2*A*	0.97	2.53	3.2884 (15)	135
C7*A*—H7*AB*⋯O4*B*^iii^	0.97	2.46	3.3796 (16)	158
C5*B*—H5*BB*⋯O4*A*^vi^	0.97	2.51	3.4141 (17)	156
C6*B*—H6*BC*⋯O1*W*^vii^	0.96	2.58	3.350 (3)	137
C7*B*—H7*BB*⋯O2*B*^iv^	0.97	2.47	3.4325 (15)	170

Symmetry codes: (i) 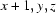; (ii) 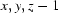; (iii) 
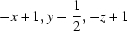
; (iv) 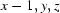; (v) 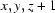; (vi) 
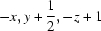
; (vii) 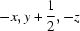.

**Fig. 1.**
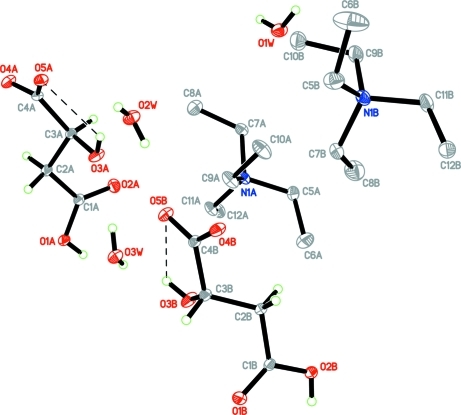
The molecular structure of (I) with atom labels and 40% probability ellipsoids for non-H atoms. The hydrogen atoms of the cations were omitted for clarity. Intramolecular interactions are shown as dashed lines.

**Fig. 2.**
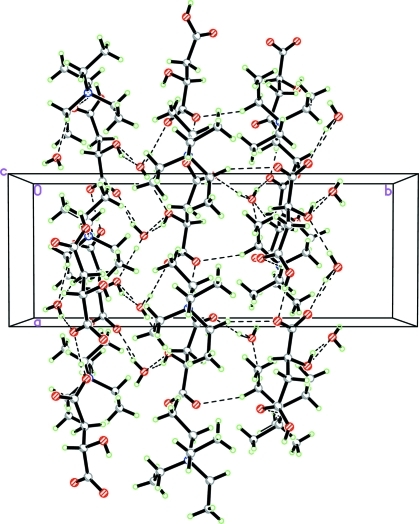
The crystal packing of (I), viewed down the c-axis showing infinite 1-D chains along the a and b-axes of the unit cell. Intermolecular interactions are shown as dashed lines.

## supplementary crystallographic information

### Comment

Previously, we have reported the formation of the tetraethylammonium L-tartarate crystal (Rahman *et al.*, 2008). In this study, we used a different anion in order to compare the interaction between the tartarate and malate ions. Generally, organic molecules contain substituents with the ability to form inter- and intramolecular hydrogen bonding. In this work, tetraethylammonium L-malate [C_2_H_5_)_4_N]^+^[C_4_H_5_O_5_]^-^, was synthesized by neutralization reaction of tetraethylammonium hydroxide with L-malic acid. Related compounds containing the same anion have been prepared (Allen *et al.*, 2006, Ying-Ying *et al.*, 2007). Tetraethylammonium hydroxide is a strong base, which easily deprotonates the carboxylic acid moiety of L-malic acid to form carboxylate anion and water as a by-product (Allen *et al.*, 2006). The reaction between tetraethylammonium hydroxide and L-malic acid forms a weak bond. It seems that the bond formed between tetraethylammonium and L-malic acid is weaker than a covalent bond but may still contribute to the achieved minimum energy configuration (Anandha *et al.*, 2008).

In the title compound I, Fig. 1, the asymmetric unit is composed of two crystallographically independent ion pairs (A and B), with similar conformations and three water molecules of crystallization. One of the water molecule (O1W) is partially occupied with a site-occupancy factor of 0.721 (5). The bond lengths (Allen *et al.* 1987) and angles are within normal ranges. Intramolecular O3A—H3OA···O5A and O3B—H3OB···O5B hydrogen bonds form *S*(5) ring motifs (Table 1) (Bernstein *et al.*, 1995). In the crystal structure, the molecules are linked together by water molecules through directed four-membered O—H···O—H···O—H interactions to form 1-D infinite chains along the *a*-axis (Fig. 2). Since the molecules are also linked into 1-D infinite chains along the *b*-axis, molecular sheets parallel to the (001)-plane are created (Fig. 2). The crystal structure is stabilized by intramolecular O—H···O (*x* 2) hydrogen bonds, intermolecular O—H···O (*x* 9) and C—H···O (*x* 10) hydrogen bonds (Table 1).

### Experimental

The synthetic procedure is similar to the previous one (Abdul Rahman *et al.*, 2008) except that L-malic acid (6.704 g, 0.05 mole) was used. Single crystals suitable for *X*-ray diffraction were obtained by slow evaporation at room temperature.

### Refinement

The H atoms bound to O1W and O2W were located from the difference Fourier map and constrained to ride on the parent atom. The hydrogen atoms of O3W were also located from the difference Fourier map and refined freely. The hydrogen of the hydroxy groups were positioned using a freely rotating O—H bond and constrained with a fixed disatnce of 0.82 Å. The rest of the hydrogen atoms were positioned geometrically and refined as a riding model. A rotating group model was used for the methyl group. One of the water molecule (O1W) is partially occupied with a site-occupancy factor of 0.721 (5). In the absence of significant anomalous dispersion effects, the Friedel pairs (6331) were averaged. Only the relative configuration is known. The highest peak (0.51 e. Å^-3^) is located 0.35 Å from H6BC and the deepest hole (-0.46 Å^-3^) is located 0.67 Å from O1W.

### Crystal data

**Table:**

C_8_H_20_N^+^·C_4_H_5_O_5_^−^·1.36H_2_O	*F*(000) = 630
*M**_r_* = 287.83	*D*_x_ = 1.248 Mg m^−^^3^
Monoclinic, *P*2_1_	Melting point: 360 K
Hall symbol: P 2yb	Mo *K*α radiation, λ = 0.71073 Å
*a* = 7.4724 (2) Å	Cell parameters from 9898 reflections
*b* = 19.9721 (5) Å	θ = 2.2–37.3°
*c* = 10.2726 (3) Å	µ = 0.10 mm^−^^1^
β = 92.481 (1)°	*T* = 100 K
*V* = 1531.64 (7) Å^3^	Block, colourless
*Z* = 4	0.45 × 0.35 × 0.32 mm

### Data collection

**Table:**

Bruker SMART APEXII CCD area-detector diffractometer	8479 independent reflections
Radiation source: fine-focus sealed tube	7551 reflections with *I* > 2σ(*I*)
graphite	*R*_int_ = 0.029
φ and ω scans	θ_max_ = 38.1°, θ_min_ = 2.2°
Absorption correction: multi-scan (*SADABS*; Bruker, 2005)	*h* = −12→12
*T*_min_ = 0.950, *T*_max_ = 0.969	*k* = −34→29
36497 measured reflections	*l* = −15→17

### Refinement

**Table:**

Refinement on *F*^2^	Primary atom site location: structure-invariant direct methods
Least-squares matrix: full	Secondary atom site location: difference Fourier map
*R*[*F*^2^ > 2σ(*F*^2^)] = 0.041	Hydrogen site location: inferred from neighbouring sites
*wR*(*F*^2^) = 0.103	H atoms treated by a mixture of independent and constrained refinement
*S* = 1.03	*w* = 1/[σ^2^(*F*_o_^2^) + (0.0604*P*)^2^ + 0.0914*P*] where *P* = (*F*_o_^2^ + 2*F*_c_^2^)/3
8479 reflections	(Δ/σ)_max_ = 0.001
373 parameters	Δρ_max_ = 0.51 e Å^−^^3^
1 restraint	Δρ_min_ = −0.46 e Å^−^^3^

### Special details

**Table:**

Experimental. The low-temperature data was collected with the Oxford Cyrosystem Cobra low-temperature attachment.
Geometry. All e.s.d.'s (except the e.s.d. in the dihedral angle between two l.s. planes) are estimated using the full covariance matrix. The cell e.s.d.'s are taken into account individually in the estimation of e.s.d.'s in distances, angles and torsion angles; correlations between e.s.d.'s in cell parameters are only used when they are defined by crystal symmetry. An approximate (isotropic) treatment of cell e.s.d.'s is used for estimating e.s.d.'s involving l.s. planes.
Refinement. Refinement of *F*^2^ against ALL reflections. The weighted *R*-factor *wR* and goodness of fit *S* are based on *F*^2^, conventional *R*-factors *R* are based on *F*, with *F* set to zero for negative *F*^2^. The threshold expression of *F*^2^ > σ(*F*^2^) is used only for calculating *R*-factors(gt) *etc*. and is not relevant to the choice of reflections for refinement. *R*-factors based on *F*^2^ are statistically about twice as large as those based on *F*, and *R*- factors based on ALL data will be even larger.

### Fractional atomic coordinates and isotropic or equivalent isotropic displacement parameters (Å^2^)

**Table:**

	*x*	*y*	*z*	*U*_iso_*/*U*_eq_	Occ. (<1)
O1A	0.64573 (10)	0.68458 (5)	1.09523 (8)	0.01788 (15)	
H1OA	0.7451	0.6730	1.0728	0.027*	
O2A	0.55148 (12)	0.61775 (5)	0.93035 (9)	0.02041 (16)	
O3A	0.27550 (11)	0.73628 (5)	0.84168 (9)	0.02079 (16)	
H3OA	0.1916	0.7510	0.7962	0.031*	
O4A	−0.03849 (11)	0.65649 (5)	1.04627 (9)	0.01975 (16)	
O5A	−0.07483 (10)	0.73468 (4)	0.88984 (9)	0.01767 (14)	
N1A	0.60300 (13)	0.67357 (5)	0.51749 (9)	0.01637 (15)	
C1A	0.52222 (13)	0.65830 (6)	1.01586 (10)	0.01443 (16)	
C2A	0.33579 (13)	0.68207 (6)	1.04454 (10)	0.01670 (18)	
H2AA	0.3433	0.7263	1.0833	0.020*	
H2AB	0.2846	0.6521	1.1072	0.020*	
C3A	0.21347 (12)	0.68474 (6)	0.92261 (10)	0.01460 (16)	
H3AA	0.2243	0.6421	0.8763	0.018*	
C4A	0.01667 (12)	0.69397 (5)	0.95518 (10)	0.01428 (16)	
C5A	0.73206 (17)	0.66565 (7)	0.40899 (12)	0.0230 (2)	
H5AA	0.6638	0.6633	0.3266	0.028*	
H5AB	0.7951	0.6235	0.4209	0.028*	
C6A	0.8688 (2)	0.72149 (11)	0.40131 (18)	0.0421 (4)	
H6AA	0.9500	0.7115	0.3340	0.063*	
H6AB	0.9346	0.7253	0.4833	0.063*	
H6AC	0.8085	0.7629	0.3817	0.063*	
C7A	0.46547 (15)	0.61822 (6)	0.49843 (12)	0.01862 (19)	
H7AA	0.4056	0.6237	0.4134	0.022*	
H7AB	0.5276	0.5756	0.4985	0.022*	
C8A	0.3248 (2)	0.61578 (9)	0.60028 (16)	0.0308 (3)	
H8AA	0.2389	0.5815	0.5778	0.046*	
H8AB	0.2652	0.6583	0.6037	0.046*	
H8AC	0.3812	0.6060	0.6839	0.046*	
C9A	0.5129 (2)	0.74197 (6)	0.51372 (13)	0.0238 (2)	
H9AA	0.4314	0.7445	0.5845	0.029*	
H9AB	0.6039	0.7760	0.5289	0.029*	
C10A	0.4094 (2)	0.75780 (8)	0.38697 (15)	0.0307 (3)	
H10A	0.3576	0.8016	0.3922	0.046*	
H10B	0.3161	0.7253	0.3723	0.046*	
H10C	0.4892	0.7565	0.3162	0.046*	
C11A	0.70013 (17)	0.66878 (6)	0.65050 (11)	0.01895 (19)	
H11A	0.7861	0.7051	0.6585	0.023*	
H11B	0.6135	0.6752	0.7171	0.023*	
C12A	0.79772 (18)	0.60338 (7)	0.67707 (12)	0.0225 (2)	
H12A	0.8461	0.6031	0.7652	0.034*	
H12B	0.8933	0.5988	0.6182	0.034*	
H12C	0.7156	0.5667	0.6647	0.034*	
O1B	1.02736 (12)	0.99945 (5)	0.57343 (9)	0.02120 (16)	
O2B	1.10569 (10)	0.93091 (5)	0.41324 (8)	0.01761 (14)	
H2OB	1.2073	0.9416	0.4385	0.026*	
O3B	0.76722 (11)	0.88088 (5)	0.67077 (9)	0.02200 (17)	
H3OB	0.6892	0.8663	0.7168	0.033*	
O4B	0.42411 (10)	0.95514 (5)	0.46248 (9)	0.01896 (15)	
O5B	0.41095 (11)	0.87999 (5)	0.62477 (9)	0.01882 (15)	
N1B	0.11875 (14)	0.93897 (5)	0.00228 (10)	0.01887 (17)	
C1B	0.99015 (13)	0.95720 (6)	0.48948 (10)	0.01417 (16)	
C2B	0.80012 (13)	0.93187 (6)	0.46378 (10)	0.01577 (17)	
H2BA	0.8044	0.8870	0.4281	0.019*	
H2BB	0.7397	0.9604	0.3993	0.019*	
C3B	0.69307 (12)	0.93078 (6)	0.58625 (10)	0.01484 (16)	
H3BA	0.7080	0.9742	0.6296	0.018*	
C4B	0.49200 (13)	0.91967 (5)	0.55639 (10)	0.01426 (16)	
C5B	−0.02184 (18)	0.99158 (7)	0.02759 (15)	0.0264 (2)	
H5BA	−0.0549	0.9877	0.1175	0.032*	
H5BB	0.0315	1.0354	0.0172	0.032*	
C6B	−0.1902 (3)	0.98789 (11)	−0.0588 (3)	0.0523 (6)	
H6BA	−0.2759	1.0197	−0.0293	0.078*	
H6BB	−0.2396	0.9436	−0.0549	0.078*	
H6BC	−0.1621	0.9980	−0.1469	0.078*	
C7B	0.26558 (17)	0.94955 (7)	0.10709 (12)	0.0215 (2)	
H7BA	0.3175	0.9935	0.0951	0.026*	
H7BB	0.2117	0.9493	0.1913	0.026*	
C8B	0.4142 (2)	0.89834 (9)	0.10931 (17)	0.0337 (3)	
H8BA	0.5074	0.9117	0.1711	0.051*	
H8BB	0.4619	0.8951	0.0242	0.051*	
H8BC	0.3678	0.8556	0.1340	0.051*	
C9B	0.03916 (19)	0.86912 (6)	0.00799 (13)	0.0232 (2)	
H9BA	−0.0503	0.8646	−0.0627	0.028*	
H9BB	0.1332	0.8369	−0.0070	0.028*	
C10B	−0.0468 (2)	0.85122 (8)	0.13452 (15)	0.0305 (3)	
H10D	−0.0843	0.8053	0.1317	0.046*	
H10E	−0.1488	0.8795	0.1459	0.046*	
H10F	0.0385	0.8576	0.2061	0.046*	
C11B	0.18992 (19)	0.94528 (6)	−0.13422 (12)	0.0224 (2)	
H11C	0.2713	0.9083	−0.1479	0.027*	
H11D	0.0901	0.9407	−0.1972	0.027*	
C12B	0.28672 (18)	1.01017 (7)	−0.16150 (13)	0.0229 (2)	
H12D	0.3146	1.0118	−0.2518	0.034*	
H12E	0.3955	1.0124	−0.1084	0.034*	
H12F	0.2113	1.0474	−0.1416	0.034*	
O1W	0.12929 (18)	0.60364 (8)	0.28868 (14)	0.0250 (4)	0.721 (5)
H1W1	0.0585	0.6171	0.2175	0.037*	0.721 (5)
H2W1	0.0955	0.5698	0.3439	0.037*	0.721 (5)
O2W	0.09626 (12)	0.80596 (5)	0.64819 (10)	0.02132 (16)	
H1W2	0.1780	0.8322	0.6271	0.032*	
H2W2	0.0171	0.8259	0.6557	0.032*	
O3W	0.61177 (12)	0.81035 (5)	0.86527 (9)	0.01997 (16)	
H2W3	0.521 (3)	0.7902 (13)	0.852 (2)	0.035 (6)*	
H1W3	0.695 (3)	0.7793 (11)	0.882 (2)	0.025 (5)*	

### Atomic displacement parameters (Å^2^)

**Table:**

	*U*^11^	*U*^22^	*U*^33^	*U*^12^	*U*^13^	*U*^23^
O1A	0.0113 (3)	0.0259 (4)	0.0164 (3)	−0.0009 (3)	0.0000 (2)	−0.0004 (3)
O2A	0.0190 (3)	0.0241 (4)	0.0180 (4)	0.0048 (3)	−0.0005 (3)	−0.0020 (3)
O3A	0.0122 (3)	0.0276 (4)	0.0226 (4)	−0.0015 (3)	0.0004 (2)	0.0107 (3)
O4A	0.0126 (3)	0.0249 (4)	0.0219 (4)	−0.0003 (3)	0.0021 (2)	0.0073 (3)
O5A	0.0131 (3)	0.0198 (4)	0.0199 (4)	0.0003 (3)	−0.0010 (2)	0.0014 (3)
N1A	0.0214 (4)	0.0148 (4)	0.0126 (4)	−0.0008 (3)	−0.0025 (3)	−0.0010 (3)
C1A	0.0128 (3)	0.0178 (4)	0.0126 (4)	0.0006 (3)	0.0002 (3)	0.0041 (3)
C2A	0.0118 (3)	0.0243 (5)	0.0140 (4)	0.0007 (3)	0.0003 (3)	−0.0003 (4)
C3A	0.0105 (3)	0.0188 (4)	0.0144 (4)	−0.0002 (3)	0.0004 (3)	0.0012 (3)
C4A	0.0109 (3)	0.0161 (4)	0.0158 (4)	−0.0020 (3)	−0.0002 (3)	−0.0015 (3)
C5A	0.0242 (5)	0.0301 (6)	0.0149 (4)	−0.0030 (4)	0.0019 (3)	0.0016 (4)
C6A	0.0367 (8)	0.0565 (11)	0.0334 (8)	−0.0218 (8)	0.0063 (6)	0.0035 (8)
C7A	0.0206 (4)	0.0170 (5)	0.0181 (5)	−0.0019 (3)	−0.0010 (3)	−0.0033 (4)
C8A	0.0261 (6)	0.0350 (7)	0.0320 (7)	−0.0060 (5)	0.0072 (5)	−0.0040 (6)
C9A	0.0357 (6)	0.0156 (5)	0.0194 (5)	0.0034 (4)	−0.0079 (4)	−0.0012 (4)
C10A	0.0425 (8)	0.0240 (6)	0.0242 (6)	0.0068 (5)	−0.0126 (5)	−0.0001 (5)
C11A	0.0268 (5)	0.0171 (5)	0.0125 (4)	0.0009 (3)	−0.0044 (3)	−0.0010 (3)
C12A	0.0267 (5)	0.0221 (5)	0.0183 (5)	0.0044 (4)	−0.0045 (4)	−0.0001 (4)
O1B	0.0184 (3)	0.0239 (4)	0.0214 (4)	−0.0047 (3)	0.0029 (3)	−0.0072 (3)
O2B	0.0115 (3)	0.0249 (4)	0.0165 (3)	−0.0017 (3)	0.0019 (2)	−0.0030 (3)
O3B	0.0127 (3)	0.0291 (5)	0.0244 (4)	0.0010 (3)	0.0026 (3)	0.0111 (3)
O4B	0.0120 (3)	0.0229 (4)	0.0219 (4)	−0.0011 (3)	0.0002 (2)	0.0063 (3)
O5B	0.0144 (3)	0.0203 (4)	0.0219 (4)	−0.0018 (3)	0.0037 (2)	0.0042 (3)
N1B	0.0236 (4)	0.0164 (4)	0.0166 (4)	−0.0034 (3)	0.0015 (3)	−0.0052 (3)
C1B	0.0129 (3)	0.0169 (4)	0.0128 (4)	−0.0018 (3)	0.0012 (3)	0.0017 (3)
C2B	0.0121 (3)	0.0202 (5)	0.0151 (4)	−0.0029 (3)	0.0021 (3)	−0.0013 (4)
C3B	0.0108 (3)	0.0179 (4)	0.0160 (4)	0.0000 (3)	0.0015 (3)	0.0014 (3)
C4B	0.0125 (3)	0.0144 (4)	0.0160 (4)	−0.0002 (3)	0.0024 (3)	−0.0017 (3)
C5B	0.0233 (5)	0.0215 (6)	0.0342 (7)	0.0000 (4)	0.0009 (4)	−0.0083 (5)
C6B	0.0329 (8)	0.0401 (10)	0.0819 (16)	0.0063 (7)	−0.0226 (9)	−0.0168 (10)
C7B	0.0243 (5)	0.0253 (5)	0.0150 (4)	−0.0036 (4)	0.0010 (3)	−0.0036 (4)
C8B	0.0309 (7)	0.0379 (8)	0.0318 (7)	0.0054 (6)	−0.0033 (5)	0.0003 (6)
C9B	0.0333 (6)	0.0172 (5)	0.0195 (5)	−0.0076 (4)	0.0046 (4)	−0.0051 (4)
C10B	0.0391 (7)	0.0288 (7)	0.0243 (6)	−0.0134 (5)	0.0080 (5)	−0.0060 (5)
C11B	0.0346 (6)	0.0185 (5)	0.0143 (5)	−0.0048 (4)	0.0018 (4)	−0.0028 (4)
C12B	0.0278 (5)	0.0203 (5)	0.0206 (5)	−0.0057 (4)	0.0026 (4)	−0.0012 (4)
O1W	0.0206 (6)	0.0304 (7)	0.0235 (7)	−0.0050 (4)	−0.0031 (4)	0.0084 (5)
O2W	0.0170 (3)	0.0205 (4)	0.0265 (4)	−0.0009 (3)	0.0011 (3)	0.0063 (3)
O3W	0.0168 (3)	0.0201 (4)	0.0231 (4)	−0.0002 (3)	0.0020 (3)	0.0046 (3)

### Geometric parameters (Å, °)

**Table:**

O1A—C1A	1.3141 (13)	O3B—C3B	1.4187 (14)
O1A—H1OA	0.8200	O3B—H3OB	0.8200
O2A—C1A	1.2215 (14)	O4B—C4B	1.2835 (14)
O3A—C3A	1.4140 (14)	O5B—C4B	1.2350 (14)
O3A—H3OA	0.8200	N1B—C5B	1.5161 (17)
O4A—C4A	1.2804 (13)	N1B—C7B	1.5184 (15)
O5A—C4A	1.2407 (13)	N1B—C9B	1.5186 (16)
N1A—C5A	1.5134 (16)	N1B—C11B	1.5262 (16)
N1A—C7A	1.5162 (15)	C1B—C2B	1.5197 (14)
N1A—C11A	1.5223 (14)	C2B—C3B	1.5200 (15)
N1A—C9A	1.5228 (16)	C2B—H2BA	0.9700
C1A—C2A	1.5127 (14)	C2B—H2BB	0.9700
C2A—C3A	1.5193 (14)	C3B—C4B	1.5366 (13)
C2A—H2AA	0.9700	C3B—H3BA	0.9800
C2A—H2AB	0.9700	C5B—C6B	1.509 (2)
C3A—C4A	1.5333 (13)	C5B—H5BA	0.9700
C3A—H3AA	0.9800	C5B—H5BB	0.9700
C5A—C6A	1.517 (2)	C6B—H6BA	0.9600
C5A—H5AA	0.9700	C6B—H6BB	0.9600
C5A—H5AB	0.9700	C6B—H6BC	0.9600
C6A—H6AA	0.9600	C7B—C8B	1.509 (2)
C6A—H6AB	0.9600	C7B—H7BA	0.9700
C6A—H6AC	0.9600	C7B—H7BB	0.9700
C7A—C8A	1.5158 (19)	C8B—H8BA	0.9600
C7A—H7AA	0.9700	C8B—H8BB	0.9600
C7A—H7AB	0.9700	C8B—H8BC	0.9600
C8A—H8AA	0.9600	C9B—C10B	1.517 (2)
C8A—H8AB	0.9600	C9B—H9BA	0.9700
C8A—H8AC	0.9600	C9B—H9BB	0.9700
C9A—C10A	1.5185 (18)	C10B—H10D	0.9600
C9A—H9AA	0.9700	C10B—H10E	0.9600
C9A—H9AB	0.9700	C10B—H10F	0.9600
C10A—H10A	0.9600	C11B—C12B	1.5163 (18)
C10A—H10B	0.9600	C11B—H11C	0.9700
C10A—H10C	0.9600	C11B—H11D	0.9700
C11A—C12A	1.5150 (17)	C12B—H12D	0.9600
C11A—H11A	0.9700	C12B—H12E	0.9600
C11A—H11B	0.9700	C12B—H12F	0.9600
C12A—H12A	0.9600	O1W—H1W1	0.9230
C12A—H12B	0.9600	O1W—H2W1	0.9242
C12A—H12C	0.9600	O2W—H1W2	0.8398
O1B—C1B	1.2297 (14)	O2W—H2W2	0.7201
O2B—C1B	1.3011 (13)	O3W—H2W3	0.80 (3)
O2B—H2OB	0.8200	O3W—H1W3	0.89 (2)
			
C1A—O1A—H1OA	109.5	C5B—N1B—C7B	105.47 (9)
C3A—O3A—H3OA	109.5	C5B—N1B—C9B	110.76 (10)
C5A—N1A—C7A	106.18 (9)	C7B—N1B—C9B	111.87 (10)
C5A—N1A—C11A	111.11 (9)	C5B—N1B—C11B	111.84 (11)
C7A—N1A—C11A	111.36 (9)	C7B—N1B—C11B	111.72 (10)
C5A—N1A—C9A	111.72 (10)	C9B—N1B—C11B	105.31 (9)
C7A—N1A—C9A	110.76 (9)	O1B—C1B—O2B	124.28 (9)
C11A—N1A—C9A	105.80 (9)	O1B—C1B—C2B	122.10 (10)
O2A—C1A—O1A	124.62 (10)	O2B—C1B—C2B	113.61 (9)
O2A—C1A—C2A	122.90 (9)	C1B—C2B—C3B	112.47 (8)
O1A—C1A—C2A	112.45 (9)	C1B—C2B—H2BA	109.1
C1A—C2A—C3A	112.13 (9)	C3B—C2B—H2BA	109.1
C1A—C2A—H2AA	109.2	C1B—C2B—H2BB	109.1
C3A—C2A—H2AA	109.2	C3B—C2B—H2BB	109.1
C1A—C2A—H2AB	109.2	H2BA—C2B—H2BB	107.8
C3A—C2A—H2AB	109.2	O3B—C3B—C2B	108.12 (9)
H2AA—C2A—H2AB	107.9	O3B—C3B—C4B	111.92 (9)
O3A—C3A—C2A	108.03 (9)	C2B—C3B—C4B	112.45 (8)
O3A—C3A—C4A	112.50 (9)	O3B—C3B—H3BA	108.1
C2A—C3A—C4A	111.89 (8)	C2B—C3B—H3BA	108.1
O3A—C3A—H3AA	108.1	C4B—C3B—H3BA	108.1
C2A—C3A—H3AA	108.1	O5B—C4B—O4B	126.45 (9)
C4A—C3A—H3AA	108.1	O5B—C4B—C3B	118.59 (9)
O5A—C4A—O4A	126.25 (9)	O4B—C4B—C3B	114.93 (9)
O5A—C4A—C3A	118.17 (9)	C6B—C5B—N1B	115.56 (13)
O4A—C4A—C3A	115.55 (9)	C6B—C5B—H5BA	108.4
N1A—C5A—C6A	114.44 (12)	N1B—C5B—H5BA	108.4
N1A—C5A—H5AA	108.7	C6B—C5B—H5BB	108.4
C6A—C5A—H5AA	108.7	N1B—C5B—H5BB	108.4
N1A—C5A—H5AB	108.7	H5BA—C5B—H5BB	107.5
C6A—C5A—H5AB	108.7	C5B—C6B—H6BA	109.5
H5AA—C5A—H5AB	107.6	C5B—C6B—H6BB	109.5
C5A—C6A—H6AA	109.5	H6BA—C6B—H6BB	109.5
C5A—C6A—H6AB	109.5	C5B—C6B—H6BC	109.5
H6AA—C6A—H6AB	109.5	H6BA—C6B—H6BC	109.5
C5A—C6A—H6AC	109.5	H6BB—C6B—H6BC	109.5
H6AA—C6A—H6AC	109.5	C8B—C7B—N1B	115.16 (11)
H6AB—C6A—H6AC	109.5	C8B—C7B—H7BA	108.5
C8A—C7A—N1A	114.88 (10)	N1B—C7B—H7BA	108.5
C8A—C7A—H7AA	108.5	C8B—C7B—H7BB	108.5
N1A—C7A—H7AA	108.5	N1B—C7B—H7BB	108.5
C8A—C7A—H7AB	108.5	H7BA—C7B—H7BB	107.5
N1A—C7A—H7AB	108.5	C7B—C8B—H8BA	109.5
H7AA—C7A—H7AB	107.5	C7B—C8B—H8BB	109.5
C7A—C8A—H8AA	109.5	H8BA—C8B—H8BB	109.5
C7A—C8A—H8AB	109.5	C7B—C8B—H8BC	109.5
H8AA—C8A—H8AB	109.5	H8BA—C8B—H8BC	109.5
C7A—C8A—H8AC	109.5	H8BB—C8B—H8BC	109.5
H8AA—C8A—H8AC	109.5	C10B—C9B—N1B	115.51 (11)
H8AB—C8A—H8AC	109.5	C10B—C9B—H9BA	108.4
C10A—C9A—N1A	114.62 (11)	N1B—C9B—H9BA	108.4
C10A—C9A—H9AA	108.6	C10B—C9B—H9BB	108.4
N1A—C9A—H9AA	108.6	N1B—C9B—H9BB	108.4
C10A—C9A—H9AB	108.6	H9BA—C9B—H9BB	107.5
N1A—C9A—H9AB	108.6	C9B—C10B—H10D	109.5
H9AA—C9A—H9AB	107.6	C9B—C10B—H10E	109.5
C9A—C10A—H10A	109.5	H10D—C10B—H10E	109.5
C9A—C10A—H10B	109.5	C9B—C10B—H10F	109.5
H10A—C10A—H10B	109.5	H10D—C10B—H10F	109.5
C9A—C10A—H10C	109.5	H10E—C10B—H10F	109.5
H10A—C10A—H10C	109.5	C12B—C11B—N1B	115.37 (10)
H10B—C10A—H10C	109.5	C12B—C11B—H11C	108.4
C12A—C11A—N1A	115.02 (10)	N1B—C11B—H11C	108.4
C12A—C11A—H11A	108.5	C12B—C11B—H11D	108.4
N1A—C11A—H11A	108.5	N1B—C11B—H11D	108.4
C12A—C11A—H11B	108.5	H11C—C11B—H11D	107.5
N1A—C11A—H11B	108.5	C11B—C12B—H12D	109.5
H11A—C11A—H11B	107.5	C11B—C12B—H12E	109.5
C11A—C12A—H12A	109.5	H12D—C12B—H12E	109.5
C11A—C12A—H12B	109.5	C11B—C12B—H12F	109.5
H12A—C12A—H12B	109.5	H12D—C12B—H12F	109.5
C11A—C12A—H12C	109.5	H12E—C12B—H12F	109.5
H12A—C12A—H12C	109.5	H1W1—O1W—H2W1	122.4
H12B—C12A—H12C	109.5	H1W2—O2W—H2W2	107.0
C1B—O2B—H2OB	109.5	H2W3—O3W—H1W3	105 (2)
C3B—O3B—H3OB	109.5		
			
O2A—C1A—C2A—C3A	−32.74 (15)	O1B—C1B—C2B—C3B	−32.15 (15)
O1A—C1A—C2A—C3A	149.15 (10)	O2B—C1B—C2B—C3B	149.04 (10)
C1A—C2A—C3A—O3A	−67.95 (12)	C1B—C2B—C3B—O3B	−68.04 (12)
C1A—C2A—C3A—C4A	167.69 (9)	C1B—C2B—C3B—C4B	167.90 (9)
O3A—C3A—C4A—O5A	14.42 (14)	O3B—C3B—C4B—O5B	14.28 (14)
C2A—C3A—C4A—O5A	136.25 (11)	C2B—C3B—C4B—O5B	136.21 (11)
O3A—C3A—C4A—O4A	−167.72 (9)	O3B—C3B—C4B—O4B	−167.58 (10)
C2A—C3A—C4A—O4A	−45.90 (13)	C2B—C3B—C4B—O4B	−45.64 (13)
C7A—N1A—C5A—C6A	173.27 (12)	C7B—N1B—C5B—C6B	−175.46 (16)
C11A—N1A—C5A—C6A	−65.49 (15)	C9B—N1B—C5B—C6B	−54.24 (19)
C9A—N1A—C5A—C6A	52.41 (15)	C11B—N1B—C5B—C6B	62.89 (19)
C5A—N1A—C7A—C8A	178.34 (11)	C5B—N1B—C7B—C8B	174.61 (12)
C11A—N1A—C7A—C8A	57.26 (14)	C9B—N1B—C7B—C8B	54.12 (15)
C9A—N1A—C7A—C8A	−60.18 (14)	C11B—N1B—C7B—C8B	−63.67 (15)
C5A—N1A—C9A—C10A	58.05 (15)	C5B—N1B—C9B—C10B	−57.11 (16)
C7A—N1A—C9A—C10A	−60.11 (15)	C7B—N1B—C9B—C10B	60.26 (16)
C11A—N1A—C9A—C10A	179.09 (13)	C11B—N1B—C9B—C10B	−178.18 (13)
C5A—N1A—C11A—C12A	−59.67 (14)	C5B—N1B—C11B—C12B	63.04 (14)
C7A—N1A—C11A—C12A	58.48 (14)	C7B—N1B—C11B—C12B	−54.94 (15)
C9A—N1A—C11A—C12A	178.89 (11)	C9B—N1B—C11B—C12B	−176.59 (11)

### Hydrogen-bond geometry (Å, °)

**Table:**

*D*—H···*A*	*D*—H	H···*A*	*D*···*A*	*D*—H···*A*
O1A—H1OA···O4A^i^	0.82	1.68	2.4977 (11)	171
O3A—H3OA···O2W	0.82	1.98	2.7296 (14)	151
O3A—H3OA···O5A	0.82	2.27	2.6853 (11)	112
O3B—H3OB···O3W	0.82	2.00	2.7435 (13)	151
O3B—H3OB···O5B	0.82	2.26	2.6837 (12)	112
O1W—H1W1···O4A^ii^	0.92	2.03	2.9354 (17)	166
O1W—H2W1···O1B^iii^	0.92	1.90	2.8018 (18)	165
O2W—H1W2···O5B	0.84	1.99	2.7969 (13)	162
O2W—H2W2···O3B^iv^	0.72	2.18	2.8961 (13)	176
O3W—H2W3···O3A	0.80 (2)	2.13 (2)	2.9169 (13)	173 (2)
O3W—H1W3···O5A^i^	0.89 (2)	1.94 (2)	2.7894 (12)	160 (2)
C2A—H2AB···O1W^v^	0.97	2.44	3.3852 (18)	165
C5A—H5AA···O1A^ii^	0.97	2.41	3.2814 (15)	149
C6A—H6AA···O1W^i^	0.96	2.59	3.296 (2)	131
C6A—H6AB···O2W^i^	0.96	2.60	3.434 (2)	146
C7A—H7AA···O1W	0.97	2.42	3.2511 (18)	144
C11A—H11B···O2A	0.97	2.53	3.2884 (15)	135
C7A—H7AB···O4B^iii^	0.97	2.46	3.3796 (16)	158
C5B—H5BB···O4A^vi^	0.97	2.51	3.4141 (17)	156
C6B—H6BC···O1W^vii^	0.96	2.58	3.350 (3)	137
C7B—H7BB···O2B^iv^	0.97	2.47	3.4325 (15)	170

Symmetry codes: (i) *x*+1, *y*, *z*; (ii) *x*, *y*, *z*−1; (iii) −*x*+1, *y*−1/2, −*z*+1; (iv) *x*−1, *y*, *z*; (v) *x*, *y*, *z*+1; (vi) −*x*, *y*+1/2, −*z*+1; (vii) −*x*, *y*+1/2, −*z*.
